# The Functions of the Skin, and Their Influence on Health

**Published:** 1855-02

**Authors:** C. H. Cleaveland


### THE FUNCTIONS OF THE SKIN, AND THEIR INFLUENCE ON HEALTH.

The human skin is designed to perform several, and very important functions: the most obvious, and perhaps the most important of which is, that of serving as a *covering* and protection to the other organs of the body, and to assist in preserving a uniform and healthy temperature.

Another, and very important function of the skin, is that which is termed the function of *perspiration*, or of letting out of the system through the pores of the skin, certain fluid and gaseous products which are no longer needed in the system, but which if retained must prove prejudicial to the health of the individual.

As the process by which the function of perspiration is performed is not fully understood, nor its importance fully appreciated by many, I propose to devote a little space to the consideration of this particular function.

Erasmus Wilson, who by physicians and physiologists, is considered good authority in regard to all that relates to the skin, says:

"To arrive at anything like an estimate of the value of the perspiratory system, in relation to the rest of the organism, I counted the perspiratory pores on the palm of the hand, and found 3,528 in the square inch. Now, each of these pores being the aperture of a little tube about a quarter of an inch long, it follows that in a square inch of skin on the palm of the hand, there exists a length of tube equal to 882 inches, or 73½ feet. Surely such an amount of drainage as 73 feet in every square inch of skin, assuming that to be the average for the whole body, is something wonderful, and the thought naturally intrudes itself—what if this drainage were obstructed? Could we need a stronger argument for enforcing attention to the skin?

"On the pulps of the fingers, where the ridges of the sensation layer of the true skin are somewhat finer than in the palm of the hand, the number of pores on a square inch exceeds that of the palm; and on the heel, where the ridges are coarse, the number of pores on the square inch was 2,268, and the length of the tube 567 inches, or 47 feet.

"To obtain an estimate of the length of tube of the *whole*

perspiratory system of the whole surface of the body, I think 2,800 might be taken as a fair average of the number of pores in the square inch, and 700 consequently as the number of inches in length.

"Now the number of square inches of surface on a man of ordinary height and build, is 2,500; the number of pores therefore, 7,000,000; and the number of inches of perspiratory tube 1,750,000; that is, 145,833 feet; or 48,600 yards, or nearly *twenty-eight miles.*

"The perspiratory system of the skin is one of the usual channels by which the excess of water is removed from the blood, and in affecting this purpose, the perspiratory function becomes a regulator of the temperature of the body.

"In health, perspiration is always taking place, even in a passive state of the body, and passes off in an imperceptible vapor, which is therefore termed *insensible* perspiration. But when the muscular system is in exercise, when chemical combination is active, and the nervous system is excited, the perspiration is no longer insensible, but becomes more or less abundant, and is then denominated *sensible perspiration.* The existence of perspiration in its sensible or insensible state, bears relation, however, not merely to the quantity of perspired fluid, but also to the state of the atmosphere.

"Thus in a close damp day when the atmosphere is already charged with moisture, it is incapable of receiving that of the skin, and the ordinary insensible perspiration becomes condensed into a sensible form.

"On the other hand when the atmosphere is dry and the body or air in motion, the moisture is carried away so rapidly, that the sensible under ordinary circumstances becomes '*insensible perspiration.*'

"The term insensible perspiration therefore, properly applies to the imperceptible evaporation when the body is at rest, or in gentle motion."

A writer in Chambers' Miscellany, while treating on this subject, says: "Throughout its whole extent, the skin consists of three layers, one over the other. The outermost, or cuticle, is an exceedingly thin substance, which may be observed to peel off when the hand is accidentally frayed, or when it is raised by a blister; the next is a layer which contains the coloring matter, giving, as the case may be, a shade from the slightest tan to the sooty black of the negro; and the third, or lowest,—the true skin,—a thick layer, which when taken off, is tanned into leather. As a whole, the skin is much more thin and delicate on one part than another,—that upon the soles of the feet and palms of the hands being, by constant use, the thickest, and most durable, and that within the lungs, the mouth, &c. being exceedingly fine, and easily injured.

"Besides their exhaling function, the pores, and other minute organs of the skin *absorb* air and moisture from the atmosphere, though with less activity than the lungs, and are therefore *inlets* as well as *outlets* to the system.

"When these pores are in a state of great openness, or relaxation from heat, or from disease, the power of absorption is greatly increased. Hence contagious diseases are more readily caught by the touch when the body is moist than when cold and dry.

"When the skin is in a proper condition and the atmosphere is pure, the vital functions suffering no impediment from external circumstances, proceed with the requisite energy, and the individual enjoys that buoyancy of spirit, which is the best criterion of health."

From the foregoing partial description of the structure and functions of the skin, it would seem to require no argument to convince all thinking beings of the great necessity for keeping that organ in a state of health, and its pores and follicles unobstructed and free in their action, as exhalents or absorbents as the case may be; yet there are many who *will not* be convinced that they should purify the entire surface of the body every day, or once a week even; and many are never thoroughly washed but once in their lives:—at their birth;—and once again after their death.

But bathing has been recommended and practised, in view of only one of the functions of the skin,—that of its power of throwing off effete matter; while its powers of *absorption*, are in a great degree overlooked: and hence the necessity of a pure *atmosphere* for the skin as well as the lungs, has seldom been attended to.

Invalids have been advised to wear underclothing of flannel, and even of the skins of animals, and other tissues impervious to the atmosphere, and have thus surrounded their bodies with an atmosphere surcharged with moisture, and filled with the heavy and offensive gases that are constantly being generated, and thrown off through the skin; to be again absorbed, and to poison the system as much as though they had been received into it through the lungs or the stomach.

A recent writer has said, "bad air is a *slow* poison," whether absorbed by the skin or by the pulmonary tissue. "That is the trouble." People go on taking it into the system, day after day, and night after night. They grow pale—their lungs suffer, the circulation is languid, they take cold readily, the chest, the stomach, the skin, becomes disordered, and a host of chronic diseases attack them. A little carbonic acid taken every day does not kill a man. It is almost a pity it did not!

If a red-hot stone destroyed instantly one man in every town daily for a week, (" or if a dirty skin would *inevitably* produce leprosy,) there might be some salvation for the nation." But when a thing is only a *slow* poison, the mass of the people are in too much of a hurry to attend to it; and those who cannot get *time* to wash the entire surface frequently, and thus keep their *twenty-eight miles* of draining ducts in a healthy, pervious condition, *will* find time to be sick and perchance to earn money to pay the doctor's bill.

The surface of the body must either be attended to, or the person cannot enjoy that state of bodily health which is necessary for a proper condition of the mind; and the expression that "cleanliness is next to godliness," has a meaning it were well for us all to understand, and heed.

I would not advise any one to become *aquatic*, but because some have considered bathing to be *the* cure of all ills, both mental and physical, is no reason why we should shut our eyes to the importance of the subject, and go through the world, sepulchres filled with all manner of impurities, with not so much as a whitened outside.

C. H. Cleaveland.

